# Supplementary material for: Circulating Tumour DNA in Advanced Melanoma Patients Ceasing PD1 Inhibition in the Absence of Disease Progression
Source: Cancers (Basel). 2020 Nov 23;12(11):3486. doi: 10.3390/cancers12113486 (PMC7700409; doi:10.3390/cancers12113486)
Supplement: Supplementary file 1 [file cancers-12-03486-s001.pdf]

Supplementary Material: Circulating Tumour DNA in Advanced Melanoma Patients Ceasing PD1 Inhibition in the Absence of Disease Progression

Lydia Warburton, Leslie Calapre, Michelle R. Pereira, Anna Reid, Cleo Robinson, Benhur Amanuel, Mel Ziman, Michael Millward and Elin Gray

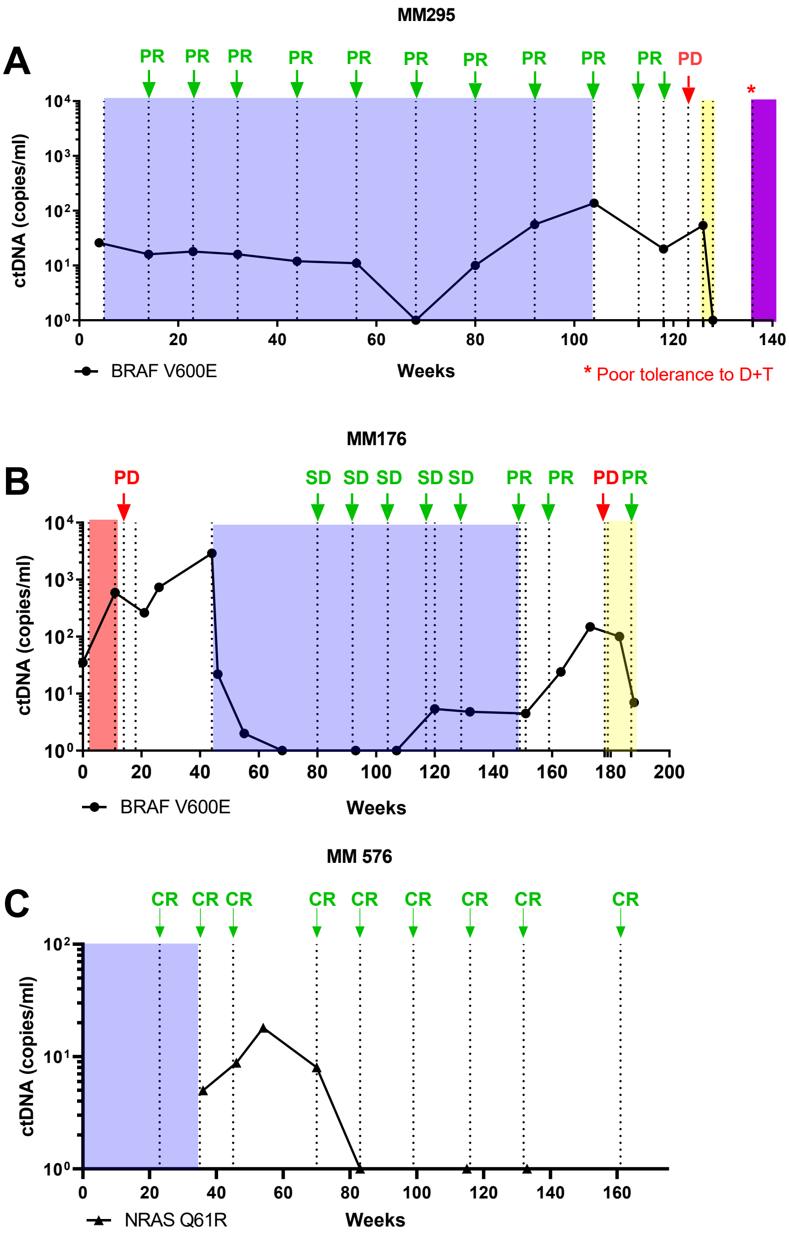

Figure S1A–S1C: Graphical representation of clinical outcomes and corresponding ctDNA over time. **S1A.** Patient MM295-BOR was PR and stopped after 2 years of anti-PD1 treatment. ctDNA level was detectable at cessation and rising 100 weeks prior to radiological confirmation of progressive disease. Progression of nodal disease was confirmed by CT/PET and biopsy five months after treatment cessation. This patient re-commenced on BRAF inhibitor treatment (Dabrafenib and Trametinib-yellow) and had an excellent response. Toxicity became unmanageable and was switched to

Vemurafenib and Cobimetinib (purple). Response is ongoing to BRAF/MEK inhibition at 52 months follow up. **S1B.** MM176 achieved PR as BOR after two years of anti-PD1 therapy. Accordingly, ctDNA reduced to undetectable levels. However, after 80 weeks of radiological disease stability, rising plasma ctDNA indicated progressive disease. CT/PET confirmed progression seven months after pembrolizumab was stopped. Treatment was switched to Dabrafenib and Trametinib. This patient died 43.4 months after the commencement of the PD1 inhibitor of disease progression. **S1C.** MM576 completed 8.3 months of pembrolizumab. He attained CR and has maintained it for the duration of follow up. Despite having detectable ctDNA at cessation of therapy, no progression eventuated.

Table S1. Detailed list of mutation types identified.

Mutations detected (n=45)	Number (%)
BRAF V600E	**7 (11)**
BRAF V600R	**2 (4)**
BRAF V600K	**2**
BRAF L597Q	**2**
BRAF K601E	**1 (2)**
BRAF V600E2	**1**
BRAF E586K	**1**
NRAS Q61K	**5 (11)**
NRAS Q61R	**5**
NRAS Q61L	**4 (9)**
NRAS G12D	**2**
TERT C250T	**2**
TERT C228T	**2**
RPS27 5'UTR C239T	**2**
GNAQ Q209L	**1**
NF1 P1851S	**1**
DPH3 C8T	**1**
RAC1 P29S	**1**
KIT L576P	**1**
TP53 R248Q	**1**
TP53 S149F	**1**

Table S2. Detailed list of blood collection time points and outcome relative to cessation.

Patient Identifier (n=38)	BOR	Mutation	Blood Collection Time Points Weeks (copies/mL)			Disease and ctDNA Outcome		TFS (mo)
			Pre cessation	At cessation	Post cessation	PD	ctDNA Detectable	
**MM490**	**CR**	**NRAS Q61R**	10 (0)	☒	☒	No	☒	2.0
MM493	CR	BRAF V600E	15 (3.6)	☒	13 (0)	No	☒	41.6
MM759	CR	TP53 S149F	6 (0)	☒	1 (0)	No	☒	26.9
MM203	CR	BRAF V600E	10 (0)	☒	☒	No	☒	42.0
MM481	CR	NRAS G12D	☒	☒	9 (0)	No	☒	33.3
MM808	CR	TERT C250T	☒	0 (0)	☒	No	☒	27.8

MM430	CR	BRAF V600K	3 (0)	☒	3 (0)	No	☒	37.0
MM642	CR	NRAS Q61L	☒	☒	5 (0)	No	☒	41.9
MM576	CR	NRAS Q61R	☒	0 (5)	10 (8.8)	No	Yes	45.1
MM512	CR	NRAS Q61R	9 (0)	☒	2 (0)	No	☒	36.6
MM669	CR	BRAF V600E	6 (0)	☒	6 (0)	No	☒	9.9
MM842	CR	NRAS Q61K	6 (0)	☒	15 (0)	No	☒	16.8
MM536	CR	NRAS G12D	☒	☒	15 (0)	No	☒	48.1
MM616	CR	TERT C250T	☒	0 (0)	13 (0)	No	☒	42.5
MM701	CR	NRAS Q61K	☒	0 (0)	☒	No	☒	26.0
MM289	CR	TP53 R248Q	13 (0)	☒	3 (0)	No	☒	51.6
MM846	CR	NRAS Q61R	☒	0 (0)	11 (0)	No	☒	23.6
MM780	CR	NRAS Q61R	1 (0)	☒	7 (0)	No	☒	15.4
MM700	CR	BRAF L597Q	10 (0)	☒	6 (0)	No	☒	23.7
MM554	CR	BRAF V600K	☒	0 (0)	☒	No	☒	43.3
MM358	CR	NRAS pQ61K	13 (0)	0 (0)	☒	No	☒	43.9
MM319	CR	BRAF V600R	6 (0)	☒	☒	No	☒	44.2
MM411	CR	GNAQ Q209L	9 (0)	☒	3 (0)	No	☒	33.4
MM237	CR	NRAS Q61K	6 (0)	☒	7 (0)	No	☒	40.9
MM239	CR	BRAF K601E	15 (0)	☒	8 (0)	No	☒	46.8
MM288	CR	RPS27 5'UTR C239T	6 (0)	☒	1 (0)	No	☒	20.9
MM748	PR	NRAS Q61K	12 (0)	☒	1 (0)	No	☒	29.7
MM545	SD	NRAS Q61L	1 (0)	☒	12 (0)	No	☒	38.0
MM798	CR	BRAF L597Q	6 (0)	☒	6 (0)	Yes	☒	11.1
MM534	CR	KIT L576P	9 (0)	☒	3 (0)	Yes	☒	16.1
MM469	CR	BRAF V600E	☒	☒	13 (1.6)	Yes	Yes	14.2
MM680	CR	DPH3 C8T	☒	0 (1.4)	12 (3)	Yes	Yes	10.1
MM549	CR	BRAF E586K	☒	☒	3 (0)	Yes	☒	15.8
MM856	CR	TERT C228T	3 (0)	☒	☒	Yes	☒	4.7
MM326	CR	TERT C228T	12	0	12	Yes	☒	21.8

			(0)	(0)	(0)			
MM630	CR	BRAF V600E	☒	0 (0)	12 (0)	Yes	☒	14.3
MM295	PR	BRAF V600E	9 (138)	☒	5 (20)	Yes	Yes	2.2
MM176	PR	BRAF V600E	16 (4.8)	☒	2 (4.5)	Yes	Yes	6.7

Abbreviations: BOR, best overall response; ctDNA, circulating tumour DNA; PD, disease progression; TFS, treatment-free survival; ☒, no blood sample available. Highlighted rows = detectable ctDNA within cessation timeframe (=/-16 weeks from cessation).
